# Exogenous N addition enhances the responses of gross primary productivity to individual precipitation events in a temperate grassland

**DOI:** 10.1038/srep26901

**Published:** 2016-06-06

**Authors:** Qun Guo, Zhong-min Hu, Sheng-gong Li, Gui-rui Yu, Xiao-min Sun, Ling-hao Li, Nai-shen Liang, Wen-ming Bai

**Affiliations:** 1 Key Laboratory of Ecosystem Network Observation and Modeling, Institute of Geographic Sciences and Natural Resources Research, Chinese Academy of Sciences, Beijing 100101, China; 2 State Key Laboratory of Vegetation and Environmental Change, Institute of Botany, Chinese Academy of Sciences, Beijing 100093, China; 3 Global Carbon Cycle Research Section Center for Global Environmental Research (CGER), National Institute for Environmental Studies (NIES), Tsukuba 305-8506, Japan

## Abstract

Predicted future shifts in the magnitude and frequency (larger but fewer) of precipitation events and enhanced nitrogen (N) deposition may interact to affect grassland productivity, but the effects of N enrichment on the productivity response to individual precipitation events remain unclear. In this study, we quantified the effects of N addition on the response patterns of gross primary productivity (GPP) to individual precipitation events of different sizes (*P*_size_) in a temperate grassland in China. The results showed that N enrichment significantly increased the time-integrated amount of GPP in response to an individual precipitation event (GPP_total_), and the N-induced stimulation of GPP increased with increasing *P*_size_. N enrichment rarely affected the duration of the GPP response, but it significantly stimulated the maximum absolute GPP response. Higher foliar N content might play an important role in the N-induced stimulation of GPP. GPP_total_ in both the N-addition and control treatments increased linearly with *P*_size_ with similar *P*_size_ intercepts (approximately 5 mm, indicating a similar lower *P*_size_ threshold to stimulate the GPP response) but had a steeper slope under N addition. Our work indicates that the projected larger precipitation events will stimulate grassland productivity, and this stimulation might be amplified by increasing N deposition.

Comprising approximately 40% of the global land cover^1^, grasslands are extraordinarily sensitive to the alteration of precipitation regimes in the context of global climate change^2^. Grassland productivity is profoundly constrained by precipitation^2^,^3^,^4^, and this constraint is characterized by features such as the total amount of precipitation and its timing, seasonal distribution, frequency, the dry spells between events, etc^5^,^6^,^7^,^8^. As an important aspect of altered precipitation regimes, the size of individual precipitation events (*P*_size_) has been predicted to dramatically change in the future; e.g., there will be a trend toward less frequent but more intense precipitation events in arid and semiarid regions^9^,^10^. Additionally, lines of evidence have indicated that the increase in atmospheric nitrogen (N) deposition due to human activities is having serious impacts on ecosystems^11^,^12^,^13^. Therefore, elucidating the interactive effects of altered precipitation regimes and N enrichment on grassland productivity is particularly important for predicting the response of ecosystems to future global climate change.

Recent studies have demonstrated that, globally, ecosystem productivity is co-limited by precipitation and N, and it is usually stimulated by the addition of water or N^14^,^15^,^16^. However, whether N enrichment could enhance productivity response to precipitation remains controversial. Studies in temperate grasslands have found that N enrichment has little effect on productivity in response to water addition^17^,^18^, and other studies have reported that the stimulation of productivity with N addition could depend on external environment conditions^15^,^16^,^19^,^20^. To date, most studies have generally focused on the effects of N addition on productivity in response to total annual precipitation (e.g., ref. 16), but availability of soil moisture is intermittent in arid and semi-arid areas. Therefore, to know how pulses of moisture following individual precipitation events affect productivity and how N addition regulate the productivity response to individual precipitation events is essential to an advanced understanding of the interactive effects between precipitation and N enrichment.

Several parameters are used in the literature to characterize the productivity-response pattern to various sizes of individual precipitation events, such as the maximum productivity response, the duration of the response, and the time-integrated amount of productivity response to a given individual precipitation event^21^,^22^. Furthermore, the productivity response is constrained by two precipitation event threshold sizes, i.e., the lower threshold (*R*^L^), which triggers the productivity response, and the upper threshold (*R*^U^), above which the response levels off^23^,^24^. Previous studies have shown that N enrichment deeply influences vegetation properties (e.g., photosynthesis or plant growth rate) and soil moisture^13^,^18^, which are also important to the determination of the above parameters^21^,^22^,^24^. However, the extent to which N enrichment affects these parameters and the effects that changed parameters will exert on the patterns of productivity in response to individual precipitation events remains unclear.

In this study, through experiments that manipulated N availability, we quantified the effects of N addition on the responses of gross primary productivity (GPP) to different sizes of individual precipitation events in a temperate steppe in Inner Mongolia, China. A multichannel automated measurement system was employed to estimate GPP at high temporal resolution, and our main objectives were as follows. First, we needed to clarify the extent to which N enrichment affected the time-integrated amount of GPP response to individual precipitation events and how the N-induced GPP response varied with the size of individual precipitation events. Second, we quantified the effects of N addition on the parameters that characterize the patterns of the GPP response to individual precipitation events, i.e., its maximum potential and duration, the lower *P*_size_ threshold initiating the GPP response and the upper threshold that achieves the maximum response (saturation). Finally, we determined how these parameters impact the total GPP response and the underlying mechanisms.

## Results

### Seasonal gross primary productivity and soil moisture dynamics

**Figure 1:**
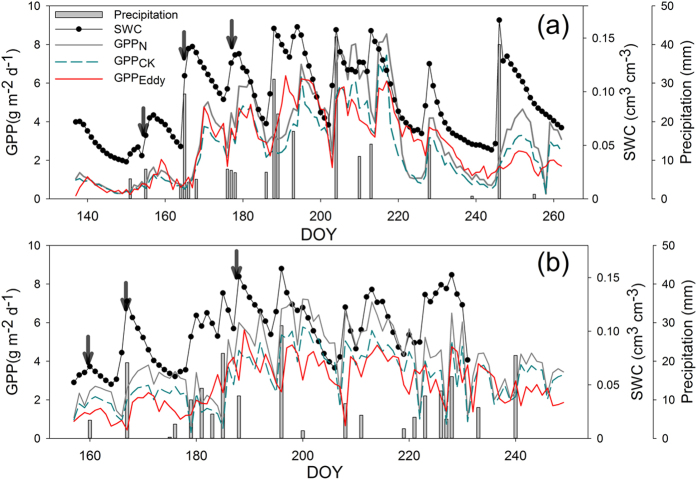
Seasonal gross primary productivity (GPP) and soil water content (SWC) dynamics of the control and N-addition treatments in 2012 (**a**) and 2013 (**b**) in a temperate grassland in Inner Mongolia, China. The six selected precipitation events are indicated by arrows above the precipitation event bars. DOY: day of year; GPP_N_: GPP of the N-addition treatment; GPP_CK_: GPP of the control; GPP_Eddy_: GPP derived from the eddy covariance system.

We found that the GPP measured by the multichannel automated measurement system in this study was highly consistent with that from an eddy covariance system (GPP_Eddy_), indicating that our GPP-estimation methods were reliable (Fig. 1). The control GPP (GPP_CK_ in terms of mean ± standard errors) during the two measurement periods (from day of year (DOY) 137 to 262 in 2012 and from DOY 157 to 249 in 2013) was 303.2 ± 25.5 g C m^−2^ (*n* = 3) in 2012 and 289.8 ± 5.7 g C m^−2^ (*n* = 3) in 2013. The GPP values of the N-addition treatments (GPP_N_ in terms of mean ± standard errors) were 395.94 ± 32.9 g C m^−2^ (*n* = 3) in 2012 and 365.75 ± 40.1 g C m^−2^ (*n* = 3) in 2013, which were significantly higher (30.6% and 26.2%, respectively) than those of the control (*n* = 3, *p* < 0.05).

**Figure 2:**
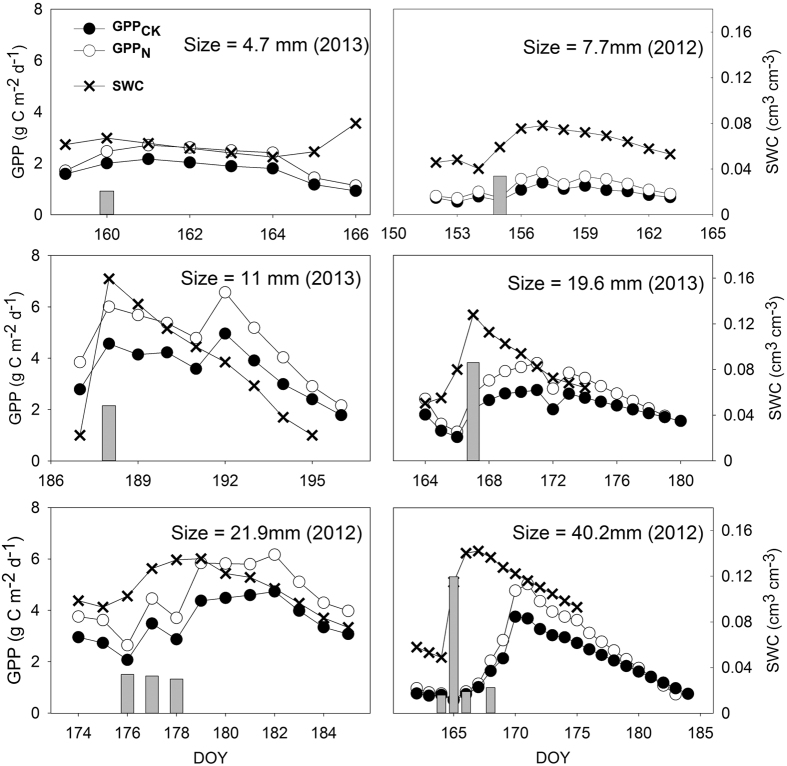
Daily GPP and SWC dynamics after different sizes of precipitation events in a temperate grassland in Inner Mongolia, China. Rainfalls occurring over consecutive days were considered to be one precipitation event (e.g., a 21.9-mm precipitation event was composed of rainfalls over three consecutive days). The *y*-axes of all of the subpanels are scaled the same for GPP, SWC, and precipitation.

Seasonally, both GPP_CK_ and GPP_N_ were closely related to the increase in soil moisture, but GPP_N_ was higher than GPP_CK_ on most days (Fig. 1). Generally, both GPP_CK_ and GPP_N_ first synchronously increased and then subsequently decreased after precipitation events. Although GPP_CK_ and GPP_N_ were more similar before rainfall, GPP_N_ was significantly higher than GPP_CK_ after rainfall, and this increase in GPP after rainfall was dependent on the size of the precipitation events (Fig. 2).

### Effects of N addition on the GPP responses to different sizes of precipitation events

**Figure 3:**
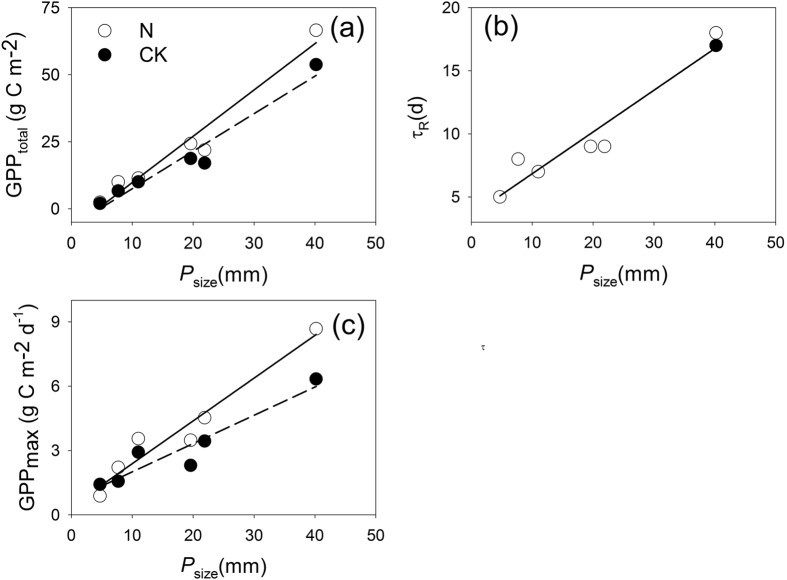
The variations in the time-integrated amount of the GPP response during the response period (GPP_total_) (**a**), the duration of the GPP response to a precipitation event (*τ*_R_) (**b**), and the maximum absolute increase in GPP induced by the event (GPP_max_) (**c**) along with the size of individual precipitation events (*P*_size_) in a temperate grassland in Inner Mongolia.

The duration of the GPP response to an individual precipitation event (*τ*_R_), the maximum absolute GPP increase induced by the event (GPP_max_), and the time-integrated amount of GPP increase during the response period (GPP_total_) all significantly linearly increased with increasing *P*_size_ in both the control and N-addition treatments (*n* = 6, *p* < 0.01, *R*^2^ ranged from 0.86 to 0.94) (Fig. 3a–c). When *P*_size_ ranged from 4.7 to 40.2 mm, GPP_total_ increased from 2.0 to 53.8 g C m^−2^ on the control and from 2.4 to 66.6 g C m^−2^ under the N-addition treatments. The GPP_max_ varied from 1.4 to 6.4 g C m^−2^ d^−1^ for the control and 0.9 to 8.7 g C m^−2^ d^−1^ for N-addition treatments, whereas the *τ*_R_ values were much more similar in the control and N-addition treatments, ranging from 5 to 18 d and 5 to 17 d, respectively.

Except for the *τ*_R_-*P*_size_ relationship (whose slopes were both 0.33), the slopes of the linear regressions of both GPP_total_-*P*_size_ and GPP_max_-*P*_size_ were steeper under the N-addition treatment than the control (Fig. 3a–c). The slope of the GPP_max_-*P*_size_ relationship in the N-addition treatment (0.21, ranging from 0.14 to 0.28) tended to be larger than that of the control (0.14, ranging from 0.09 to 0.21) (*n* = 6, *p* = 0.09). Although the slope of the GPP_total_-*P*_size_ regression (1.72, ranging from 1.32 to 2.36) did not significantly increase due to N addition (*n* = 6, *p* = 0.22), it was still much larger than that of the control (1.39, ranging from 1.06 to 1.93). However, the lower precipitation event threshold size (*R*^L^) required to induce a GPP response, i.e., the *P*_size_ intercept of the GPP_total_-*P*_size_ regression, was much more similar in both the N-addition treatments (4.92 ± 1.85 mm) and the control (5.23 ± 1.88 mm) (*p* > 0.1). The upper precipitation event threshold (*R*^U^), at which GPP response leveled off, was not observed in this study.

**Figure 4:**
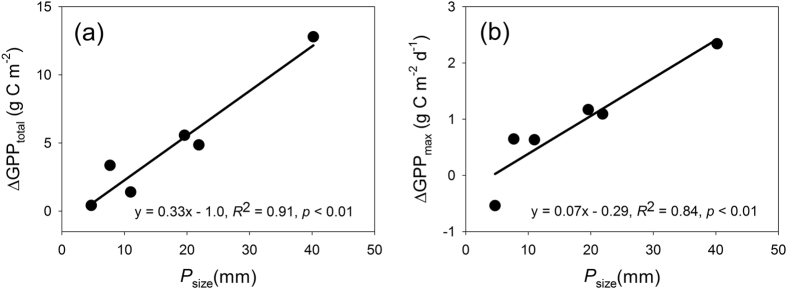
Variations of the differences in GPP_total_ (ΔGPP_total_) and GPP_max_ (ΔGPP_max_) between the control and N-addition treatments with the size of precipitation events (*P*_size_) in a temperate grassland in Inner Mongolia.

The difference in GPP_total_ between the N-addition treatments and the control increased significantly with increasing *P*_size_ (Fig. 4a), while the difference of GPP_max_ also increased (Fig. 4b). There was no remarkable difference in *τ*_R_ (*n* = 6, *p* > 0.1), indicating that the N-induced stimulation of GPP_total_ is mainly due to the increased GPP_max_.

### Leaf area index and foliar N content

**Figure 5:**
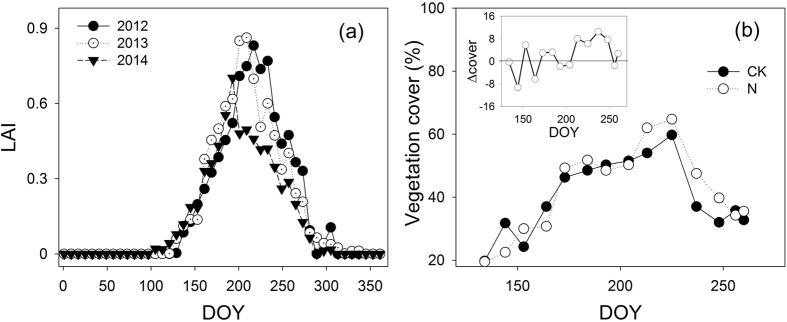
Leaf area index (LAI) over three years (**a**) and vegetation cover in 2014 (**b**). The panel inset in (**b**) shows the difference in vegetation cover (Δcover) between the N-addition (N, empty circle) and the control (CK, solid circle) treatments in 2014. LAI was derived from normalized difference vegetation index (NDVI) data from the Moderate Resolution Imaging Spectroradiometer (8-d time resolution and 250-m spatial resolution).

The leaf area index (LAI) in 2012, 2013, and 2014 exhibited a hump-shaped pattern of seasonal variation (Fig. 5a). LAI was very similar among years in the early growing season (from DOY130 to 190) but differed considerably throughout the rest of the growing season (*n* = 13, *p* = 0.04), being much lower in 2014 due to a long dry spell (21 days) (Fig. 5a). Lacking vegetation cover data for each treatment in 2012 and 2013, we used the vegetation cover data obtained in 2014 to approximate the status of the vegetation cover in 2012 and 2013 based on the LAI similarity in the early growing season (i.e., the study period), and then we evaluated the vegetation cover differences between the treatments in 2012 and 2013. The vegetation cover increased at first and reached its peak in mid-August (Fig. 5b). There was no difference in vegetation cover between the N-addition treatments and the control during the early growing season (*n* = 8, *p* = 0.91), but a larger difference appeared from the middle to the end of the growing season (*n* = 5, *p* = 0.41).

**Figure 6:**
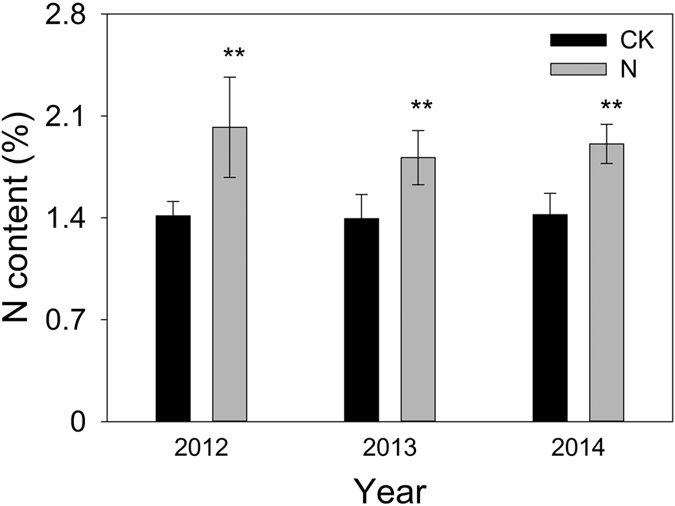
Comparison of the foliar N content among different years and treatments. An asterisk above the error bars indicates a significant difference between treatments (*p* < 0.001). Values showed in the figure are averaged with standard errors from 8 plots of each treatment.

No significant (the control) or only slight (the N-addition treatments) inter-annual variations in foliar N content were observed across the three years (Fig. 6). Foliar N content in the N-addition treatments was higher than that of the control (*n* = 8, *p* < 0.001); i.e., foliar N content in the N-addition treatments was 43%, 30%, and 34% higher than that in the control in 2012, 2013, and 2014, respectively (Fig. 6).

## Discussion

The time-integrated amount of the GPP response to an individual precipitation event (GPP_total_) greatly increased due to N addition in the temperate steppe of Inner Mongolia, and this stimulation increased with the size of the precipitation event. Previous studies have shown that exogenous N addition boosts the productivity response to the amount of annual precipitation^15^,^16^, and our observations provide evidence that N-induced stimulation of productivity could occur in response to an individual precipitation event. In arid and semi-arid ecosystems, such as the steppe in this study, productivity is primarily limited by water availability^25^,^26^,^27^, so ecosystems are only limited by nitrogen when the soil water content reaches a critical level^28^. Thus, we may postulate that N addition-induced stimulation of the GPP response to individual precipitation events may be more likely when there is a relatively long-lasting period of ample soil moisture. Soil moisture pulses due to larger precipitation events that percolate deep into the soil profile may serve as an important water source that can be maintained for much longer periods^29^,^30^, leading to more benefits of plant growth (GPP) from greater N release, so the stimulation of GPP_total_ due to N addition increased with *P*_size_. It has been predicted that precipitation events will tend to be larger in size but fewer in number in the context of global climate change^10^, which may alleviate the water limitation imposed on vegetation, especially in arid and semi-arid environments, and thus promote plant growth (GPP)^29^,^30^,^31^. This stimulation of productivity under altered precipitation regimes will likely be further augmented in the context of increasing N deposition in the future.

For any individual precipitation event, the increase in GPP_total_ by N enrichment could be primarily ascribed to the enhancement of the maximum absolute GPP response induced by the event (GPP_max_) rather than the duration of the GPP response (*τ*_R_). Under the given soil moisture conditions due to an individual event, N enrichment will stimulate GPP_max_ in two ways. Firstly, the photosynthetic capacity may be enhanced by increased foliar N content, and previous studies have shown that the increase in foliar N content resulting from N enrichment is one of the main reasons for the enhancement of photosynthesis^32^,^33^,^34^,^35^. Secondly, N enrichment relieves N limitation in ecosystems and subsequently increases plant growth and vegetation cover^18^, thus facilitating higher carbon sequestration per unit area. Our results showed that N addition did boost foliar N content relative to the control, whereas leaf area index was much more similar in both the control and N-addition treatments. Due to the relatively low temperatures during the study period (i.e., the early growing season), plants grew slowly, so a significant increase in vegetation cover due to N addition was not observed. However, obviously higher vegetation cover was observed in the N-addition treatments than in the control during the warmer mid-late growing season, indicating that an increase in vegetation cover might also contribute to stimulating GPP_max_. Previous studies have revealed that N enrichment could affect soil moisture by impacting vegetation cover^18^, and it has been indicated that *τ*_R_ is constrained by the duration of higher soil moisture^22^. Therefore, N enrichment could affect *τ*_R_ by modulating soil moisture resulting from rainfall. Nevertheless, there was no striking difference in vegetation cover between the control and N-addition treatments, leading to a similar *τ*_R_.

N enrichment had minor impacts on the lower *P*_size_ threshold (*R*^L^) that triggers a detectable GPP-response. Previous studies have demonstrated that the productivity response starts at an approximately 5-mm *P*_size_ threshold in typical steppes of Inner Mongolia^36^,^37^, which is similar to the observation in this study. As plant growth is primarily constrained by water availability, it appears that the steppe is more limited by soil moisture rather than N availability when the *P*_size_ is relatively small, e.g., less than 5 mm, implying that N enrichment has a minor or negligible impact on *R*^L^. When the *P*_size_ was larger than *R*^L^, GPP_total_ increased linearly with *P*_size_ in both treatments because of the increasing relief from water limitation and hence a greater plant response^22^. Previous studies have proposed that a plant’s physiological activity cannot exceed some maximum value due to functional or structural constraints, and furthermore, soil moisture would be saturated after precipitation events of a certain size^24^. Therefore, there should be an upper *P*_size_ threshold (*R*^U^), above which no additional GPP response occurs. *R*^U^ was not observed in this study because such sizes of individual precipitation events, especially with a *P*_size_ larger than 50 mm, were not observed, but *R*^U^ deserves further examination in the future.

In conclusion, based on a field experiment consisting of N addition and continuous measurements of net ecosystem CO_2_ exchange with a multichannel automated measurement system, we quantified the effects of N addition on the responses of GPP to individual precipitation events in a temperate steppe in Inner Mongolia, China. Our results showed that N enrichment increased the time-integrated amount of the GPP response to an individual precipitation event through the enhanced maximum absolute GPP response after the event, and the stimulation of GPP induced by N addition increased with the size of the precipitation events (*P*_size_). N enrichment rarely affected the lower *P*_size_ threshold for inducing a detectable GPP-response. Our work has important implications for theoretical modeling to obtain an advanced understanding of the response of productivity to different sizes of precipitation events and increased N deposition in grassland ecosystems.

## Methods

### Study site

This study was conducted at the Duolun Restoration Ecology Research Station of the Institute of Botany, Chinese Academy of Sciences, which is located in a typical steppe in Inner Mongolia, China (42°02′N, 116°117′E; 1,324 m a.s.l.). The dominant species in this grassland include *Stipa krylovii* Roshev., *Artemisia frigid* Willd., *Potentilla acaulis* L., *Cleistogenes squarrosa* (Trin.) Keng., *Allium bidentatum* Fisch. ex Prokh., and *Agropyron cristatum* (L.) Gaertn. Mean annual precipitation (MAP) is approximately 385.5 mm with the majority (over 80%) falling in the growing season from May to September. Annual mean temperature is 2.1 °C with the highest monthly mean occurring in July (18.9 °C) and the lowest in January (−17.5 °C). The soil type is chestnut; the soil pH value is 7.12; and the mass-based nitrogen and phosphorus content is 0.17% and 0.28%, respectively. The length of the growing season is approximately 150 d, and the average plant height is 0.4 m. During the two years of the experimental period, both the amount of precipitation (285 mm in 2012 and 262 mm in 2013) and the median precipitation event size (6.3 mm in 2012 and 7.7 mm in 2013) were similar. The aboveground biomass estimated by the harvest method was 151.3 g m^−2^ in 2012 and 143.6 g m^−2^ in 2013, respectively, indicating that the climate and vegetation cover did not differ greatly between 2012 and 2013.

### Design of the manipulative experiments

The experiment employed a complete randomized block design with two treatments, including a control (0 g N m^−2^ yr^−1^) and one N-addition level (10 g N m^−2^ yr^−1^), the rate of which is similar to the critical threshold for the N-induced increase in aboveground biomass^38^. A total of four parallel blocks (each block consisted of 4 3-m × 4-m plots) were established. Two of the four plots in each block were set as control plots, and the remaining two plots were used for the N addition treatment. There were buffer zones of at least 3 m between blocks and intervals of 2 m between the plots within each block. The manipulative N-addition experiments began in 2012 and have continued to this day.

Urea (CO(NH_2_)_2_) was scattered on the N-addition plots twice before the first rainfall of each month during the growing season, from May to June, each year (2012 and 2013). If there were no rains in the first half of each month, the urea solution would be added to the N-addition plots, and an equal amount of water would be added to the control plots. However, during our two-year experiment period, this situation did not occur. N addition was performed on May 11 and June 3 in 2012 and May 3 and June 5 in 2013.

### Measurements of gross primary productivity and meteorological variables

We measured net ecosystem CO_2_ exchange between the atmosphere and grassland with a multichannel automated measurement system developed by Dr. Liang at the National Institute for Environmental Studies of Japan. The system is comprised of 12 transparent chambers and a control box, and six chambers were used in this study, i.e., three for the control and three for the N-addition treatments. The main components of the control box are an infrared gas analyzer (IRGA, Li-840, Li-Cor Inc., Lincoln, USA) and a data logger (CR3000, Campbell Scientific, Inc., Logan, UT, USA), and detailed diagram of this multichannel automated measurement system can be found in ref. 22. During the measurements, 12 chambers are sequentially closed by a homemade relay board controlled by the data logger and then the air in the closed chamber is circulated through the IRGA by a microdiaphragm pump (CM-50, Enomoto Ltd., Tokyo, Japan). The sampling period for each chamber is 150 s (i.e., it takes 0.5 h to measure all of the 12 chambers), and the data logger monitors the CO_2_ concentration output signals from the IRGA at a rate of 1 Hz and records their averages at 10-s intervals. To remove the interference from impure air and erratic air pressure, we excluded the data from the first 10 s and the last 10 s. We obtained one measurement from each chamber in half an hour, and the net ecosystem exchange of CO_2_ (NEE) was calculated from the CO_2_ exchange rate during the measurement periods (i.e., 130 s):


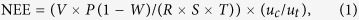


where *V* is the volume of the chamber (m^3^); *P* is the air pressure (Pa); *W* is the water vapor mole fraction; *R* is the universal gas constant (8.314 Pa m^3^ mol^−1^ K^−1^); *S* is the base area of the chamber (m^2^); *T* is the air temperature in the chamber (K); and *u*_c_/*u*_t_ is the rate of increase in the CO_2_ mole fraction (mmol mol^−1^ s^−1^) in the chamber calculated by the least squares method.

Because nighttime NEE only represents ecosystem respiration (*R*_e_), we used the relationship between nighttime NEE and soil temperature to estimate daytime *R*_e_ (*R*_ed_)^39^. Furthermore, soil water content (SWC) was also considered when evaluating the *R*_ed_ because our study was carried out in a dry environment^40^. In this study, *R*_ed_ was finally estimated as follows:


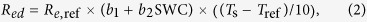


where *R*_e,ref_ is the ecosystem respiration at the reference temperature (*T*_ref_, 10 °C) and optimal soil moisture; *T*_s_ is the soil temperature; *b*_1_ and *b*_2_ are constants evaluated from the relationship between nighttime *R*_e_ and temperature and SWC.

Finally, GPP was indirectly estimated as follows:


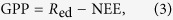


where the negative NEE denotes a carbon flux into the steppe, and a positive NEE denotes the reverse. Because our GPP estimation methods are similar to those from an eddy covariance system, which we also have in the same field experiment, we compared the results between the two systems to evaluate the uncertainty of the GPP estimation in this study.

There are two thermocouples in each chamber to measure the air temperature and the soil temperature (at a depth of 5 cm), which are recorded by a data logger with the same time resolution as that of the CO_2_ flux. Volumetric soil water content at the depths of 5 cm, 20 cm, and 40 cm were measured and calculated simultaneously at half-hourly and daily intervals with a meteorological measurement system near the treatments. Finally, we acquired daily GPP and soil water content data from DOY 137 to DOY 262 in 2012 and from DOY 157 to DOY 249 in 2013. The missing growing season data were mostly due to the rough conditions when the experiments must be suspended or the failure of the multichannel automated measurement system.

Leaf samples of five dominant species in each plot were collected in mid-August to determine foliar N content, and the N content of the five species was averaged for each plot. Vegetation cover for the two treatments was estimated by means of SamplePoint software^41^ from the quadrat photos taken every week in 2014. Vegetation cover data were missing because the quadrat photos were not available in 2012 and 2013.

### Data analysis

In the present study, an individual precipitation event was defined as a series of one or more consecutive days of rainfall followed by at least one day without rainfall.

The GPP observed from 3 transparent chambers was averaged to evaluate the GPP for each treatment. We denoted GPP prior to the precipitation event as GPP_base_, which was averaged over the three days before the event to better represent pre-rainfall GPP. Then, we evaluated the GPP response to an individual precipitation event in both the control and N-addition treatments in terms of its duration, maximum, and time-integrated amount. (1) The duration of the GPP response (*τ*_R_) was calculated as the number of days required for GPP to return to its pre-precipitation event level, i.e., the GPP_base_. If the daily GPP did not reach the GPP_base_ due to the occurrence of the next precipitation event, we used a linear extrapolation method for the estimation. (2) The maximum absolute GPP increase (GPP_max_) induced by the precipitation event was calculated as the maximal difference between daily GPP after rainfall and GPP_base_. (3) The time-integrated amount of the GPP response during the response period (duration) (GPP_total_) was calculated as


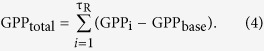


where subscript *i* is the *i*th day after the occurrence of a precipitation event.

Based on the regression between GPP_total_ and *P*_size_, we also evaluated the lower and upper event size threshold that stimulated a detectable GPP-response or at which no additional gain in the GPP response would result from the increasing size of the event, e.g., the intercept of a linear function (lower threshold) or the critical thresholds of a logistic curve (lower and upper thresholds).

The GPP response to an individual precipitation event may be confounded by other factors, e.g., antecedent soil water prior to the precipitation event^42^, leaf area index, and photosynthetic capacity at different growth stages^21^,^24^,^43^. To precisely evaluate the GPP response to an individual precipitation event, several criteria must be satisfied. First, antecedent SWC should be very low (mostly lower than the average SWC, i.e., 0.088 cm^3^ cm^−3^ in this study), and the inter-pulse period should be longer than 5 days to minimize the effect of the previous precipitation events, which may shadow or confound the GPP response to the current precipitation event of interest. Second, we normalized the GPP-response (i.e., GPP_max_, GPP_total_, but not *τ*_R_ because *τ*_R_ is mostly affected by the duration of SWC) according to LAI. LAI was evaluated by normalized difference vegetation index (NDVI) data with models developed by ref. 44 (LAI = 0.106e^4.064NDVI^, *R*^2^ = 0.94). NDVI data were from the Moderate Resolution Imaging Spectroradiometer (MODIS) with 8-d time resolution and 250-m spatial resolution (http://daac.ornl.gov/MODIS/modis.html). Finally, the GPP response to a precipitation event might vary at different growth stages because of varying photosynthetic capacities (assuming a stronger response in the middle of the growing season than early or late in the growing season), and for the purpose of comparison, we chose the neighboring precipitation events to minimize the possible effects of vegetation phenology.

In total, six independent precipitation events from DOY 155 to 188 were selected for the related analysis (7.7, 40.2, and 21.9-mm precipitation events on DOY 155, 164, and 176 in 2012 and 4.7, 19.6, and 11-mm precipitation events on DOY 159, 168, and 188 in 2013). Hence, our results only reflected the GPP response during the early growing season.

### Statistical analysis

Normality of data was tested by the Kolmogorov-Smirnov test. One-way ANOVA was employed to estimate the differences in the variables of interest (e.g., GPP, GPP_total_, LAI, vegetation cover, foliar N content, etc.) between the two treatments or the different years. Comparison of slope differences between the two treatments was performed using the *R* software package (version 3.1.2, an open source software program), and the slope comparison was implemented by the “smart” package with “slope.com” command. A *t*-test was used to estimate whether these differences were significant, and the significance level was set at 0.05.

## Additional Information

**How to cite this article**: Guo, Q. *et al.* Exogenous N addition enhances the responses of gross primary productivity to individual precipitation events in a temperate grassland. *Sci. Rep.* **6**, 26901; doi: 10.1038/srep26901 (2016).
